# Data Governance in the Dairy Industry

**DOI:** 10.3390/ani11102981

**Published:** 2021-10-15

**Authors:** Roger Cue, Mark Doornink, Regi George, Benjamin Griffiths, Matthew W. Jorgensen, Ronald Rogers, Amit Saha, Kyle Taysom, Victor E. Cabrera, Steven R. Wangen, Liliana Fadul-Pacheco

**Affiliations:** 1 Department of Animal Science, McGill University, Montréal, QC H9X 3V9, Canada; roger.cue@mcgill.ca; 2 VES-Artex, Chippewa Falls, WI 54729, USA; markd@ves.co; 3 Nalytix LLC, Hartland, WI 53029, USA; regi.george@nalytix.com; 4 Office of Legal Affairs, University of Wisconsin-Madison, Madison, WI 53706, USA; ben.griffiths@wisc.edu; 5 USDA—ARS Livestock Behavior Research Unit, West Lafayette, IN 47907, USA; matthew.jorgensen@usda.gov; 6 Prairie Estates Genetics, Middleton, WI 53562, USA; rrogers@prairieestatesgenetics.com; 7 FoGS Global Research and Consultancy Centre, Ahmedabad 382010, Gujarat, India; fogsconsulting@outlook.com; 8 Dairyland Laboratories Inc., Arcadia, WI 54612, USA; ktaysom@dairylandlabs.com; 9 Department of Animal and Dairy Sciences, University of Wisconsin-Madison, Madison, WI 53706, USA; vcabrera@wisc.edu; 10 American Family Insurance Data Science Institute and Wisconsin Institute for Discovery, University of Wisconsin–Madison, Madison, WI 53715, USA; srwangen@wisc.edu

**Keywords:** data security, data privacy, data sharing, Farmers Bill of Rights

## Abstract

**Simple Summary:**

There are no existing laws that regulate data usage in the dairy industry in the U.S. Therefore, challenges regarding data management, availability, use, and security are a growing concern. These concerns are especially important in the light of the growing dependency and monetization of such data. Although some codes of practices have been published, these are voluntary, which makes them legally vulnerable and without guarantees under the law. Thus, in this paper, we propose creating a Farmers Bill of Rights to standardize data governance and data regulations and to promote an equitable relationship of dairy data governance between farmers, industry professionals, and companies.

**Abstract:**

Data governance is a growing concern in the dairy farm industry because of the lack of legal regulation. In this commentary paper, we discuss the status quo of the available legislation and codes, as well as some possible solutions. To our knowledge, there are currently four codes of practice that address agriculture data worldwide, and their objectives are similar: (1) raise awareness of diverse data challenges such as data sharing and data privacy, (2) provide data security, and (3) illustrate the importance of the transparency of terms and conditions of data sharing contracts. However, all these codes are voluntary, which limits their adoption. We propose a Farmers Bill of Rights for the dairy data ecosystem to address some key components around data ownership and transparency in data sharing. Our hope is to start the discussion to create a balanced environment to promote equity within the data economy, encourage proper data stewardship, and to foster trust and harmony between the industry companies and the farmers when it comes to sharing data.

## 1. Introduction

New emerging technologies that are available for dairy farms come with an increase of data availability. The use of this data can help improve the production performance, animal welfare, animal health, and overall sustainability of the dairy industry [1]. As such, the collection, aggregation, and analysis of data are foreseen to transform the decision-making process on farms [2]. Nevertheless, the increase of data availability and data collection has also generated some challenges, such as the need to manage all the data, the need to guard against the potential misuse of data, and to maximize the utility of these data [3]. In general, the increased volume, importance of, and reliance on, such data has increased interest in the proper stewardship of this data to ensure that its use and storage is performed in a way that not only maximizes effectiveness but also ensures that the data is kept out of harm’s way. To achieve this, organizations often codify rules and procedures that ensure the safety, integrity, and efficiency of data storage and access. This process, commonly known as data governance (see References [4,5,6]), defines those guidelines that guide the actions (data stewardship) required to protect that data. Typically, these processes are implemented within organizations; in contrast our objective is to define a set of uniform, mutually beneficial principles that guide the exchange of data between entities (be they companies, farms, institutions, etc.). 

To deal with this growing concern, codes of conduct around data governance and stewardship have been developed in different parts of the world. The main objectives of these agricultural codes of practice are to build trust between the farmers and agribusiness, *ideally* with a contractual agreement, and raise awareness of the terms and conditions of data sharing [7]. Among these data codes of conduct are the United States American Farm Bureau’s “Privacy and Security Principles for Farm Data” [8], the New Zealand “Farm Data Code of Practice” [9], the Australian “Farm Data Code” [10], and the European Union “Code of Conduct on Agricultural Data” [11]. One of the characteristics that these codes of practice have in common, and maybe the most remarkable, is that they are voluntary; as such, they can only serve as guidelines for a formal contractual agreement. However, van der Burg et al. [7] mentioned that the idea of the contractual agreement by itself may not be enough to allay the concerns of the data generators and encourage participation of the farmers in a data economy. The problem with the contract is that it generates limited trust. van der Burg et al. [7] referred to the work of bioethicist Onora O’Neill to explain the limited trust of contract agreements between parties with different powers, since they differ in expertise and knowledge; a similar dichotomy occurs between agribusiness (i.e., the digital experts) and the farmers (i.e., the nondigital experts). The limited trust due to the knowledge disparity, also known as the data inequality, is currently a global technological issue, and it has been recently reviewed and discussed by “Data Fairness, an exploration of key data inequality trends, their causes and the available tools and ideas to solve them” produced by the MIT Technology Review Insights in partnership with Omidyar Network [12]. Briefly, this review focused on the inequality of the data economy, which is dominated by a small number of companies. Governments and citizens that are essential contributors to the data economy are not part of the development nor the profits of it; as data has become a resource, the value of it needs to be accurately and fairly estimated. As such, more complex tools are needed to understand the dynamics and properties of data [12].

To avoid the possible negative consequences of this unbalanced relationship, all the codes of practices referenced above advise that it is important to promote transparency within said relationship. This can be primarily achieved using plain language in the terms and conditions of the contract, for example, in describing the purposes for which the data is being collected, used, or shared; how the data will be used and managed; what possible risks may affect the farmers; information about service termination, etc. [8,9,11,13]. In addition, the American Farm Bureau Federation (AFBF) [8] and the Data Fairness [12] suggest that educating farmers and citizens to better understand their rights and responsibilities can also be beneficial in establishing trust. 

In addition to addressing the data imbalance, transparency is also a necessary condition in order for farmers to fully participate in the data economy (either by monetizing data or purchasing derivatives). This requires access to agreements they can trust and understand. Transparency in agreements that address data sharing and data governance policies is critical to establish the trust needed to ensure their participation. In an effort to bring these issues into the forefront, this commentary article focuses on two objectives: (1) to discuss the current state of data governance in the dairy farm industry, emphasizing the situation in the United States, and (2) to raise questions and kindle a discussion about possible solutions regarding data governance and data stewardship in dairy farming. 

## 2. Background and Procedure 

This commentary paper is the result of a collaborative effort from the University of Wisconsin-Madison Dairy Brain team and the Dairy Brain Coordinate Innovation Network (CIN) (https://dairybrain.wisc.edu/coordinated-innovation-network/, accessed on 13 March 2021). The Dairy Brain CIN is an industry-wide stakeholder group discussing data challenges in the dairy industry and guiding the Dairy Brain team in their research. The Dairy Brain CIN was established in September 2019, succeeding an initial project Advisory Committee. It currently enlists more than 100 members composed of dairy farmers from across the globe, dairy industry professionals, policymakers, and academics who provide insights and guidance to the University of Wisconsin-Madison Dairy Brain Project. 

The Dairy Brain CIN has committed to publishing documents about several topics, including data governance, best practices for data collection and communication, creating value from farm data, strategies for data utilization and monetization, and related issues. As part of this effort, the Dairy Brain CIN has already published five articles in Hoard’s Dairyman magazine between February and May 2020 [14,15,16,17,18]. After these publications, the group entertained open forum discussions through its website and social media platforms. These and internal discussions identified those topics with the greatest need for more in-depth discussion. Among those needs were the sustainable adoption of decision support tools, which was recently published [19], and data governance, which is addressed in this article.

Once the main topic was loosely defined, an invitation was extended to all Dairy Brain team and CIN members to gather their interest and willingness to collaborate and coauthor this manuscript. The listed authors of this article are the ones who responded positively to the call and contributed throughout the process. 

An outlined draft document was shared among all participants and remained open for editing, contribution, suggestions, and commentary during the entire process. The process involved discussion focus groups, meetings, constant communication between the Dairy Brain CIN and the Dairy Brain team, and prominent feedback among all the participants. The Dairy Brain team took the lead on processing and updating the manuscript, but the whole group had the opportunity to provide input into the process at any time. A highly inclusive environment was promoted to ensure a truly collaborative effort that represents the consensual opinions of all the authors.

## 3. Current State of Data Governance in the Dairy Farm Industry

Data sharing is a common practice within the dairy farm industry (e.g., Council on Dairy Cattle Breeding (CDCB) and Dairy Herd Improvement Associations (DHIA)). Lately, there has been an increase in the amount of data generated by farms, primarily due to the increased adoption of automated systems and technologies such as sensor arrays. With this, there have been growing concerns regarding the ownership and usage of data generated on farms, as there are currently no existing laws that address it [20,21,22]. It has been suggested that a federal policy should be put in place to control data practices in the agricultural sector [22]. The most recent attempt to address the topic was five years ago, when the AFBF published the (voluntary) “Privacy and Security Principles for Farm Data” [8]. Since that time, there has been little advancement to address data ownership, data privacy, and data security in dairy settings. The specific statement from the AFBF policy on ownership states that: “We believe farmers own information generated on their farming operations. However, it is the responsibility of the farmer to agree upon data use and sharing with the other stakeholders (...)” [7]. To get a better understanding of the statement, Janzen [20] broke the statement into three parts: “(1) the farmers own the data created on their farms; (2) there are multiple stakeholders that may be interested in the farmer’s data when using online platforms; and (3) farmers are responsible for making sure the data they upload is theirs or used by permission”.

From a legal perspective, data ownership is difficult to address because of characteristics of the data: the farm data are intangible, irreplaceable, and nonrival [22]; these characteristics largely prohibit the reapplication of existing laws to this use case. The federal law recognizes ownership for physical property, and farm data is viewed as intangible, due to its digital nature [20,22]. Therefore, the only remaining possible legal classifications for protecting farm data ownership are to classify it as intellectual property. Intellectual property could be protected via patents or copyrights or considered as trade secrets (i.e., formulas or methods) [20,22]. However, even with this classification, farmers are limited in their capacity to address data ownership. In 2019, a study in Australia investigated the main concerns of data sharing and found that the lack of legal standards and lack of transparency about the terms of use in data licenses, ownership of data, and data sharing were the most frequently reported concerns [23]. This was also found in the results obtained from a survey done by the Dairy Brain project at the University of Wisconsin-Madison recently (2021), where 59% of farmers indicated that they have not signed a data sharing agreement during the past 5 years. In addition, 22% farmers indicated that they do not know if they have signed data sharing agreements during the past 5 years (unpublished data). Even though, eventually, all farmers share their data in one way or another. Thus, these concerns are viewed as consistently important worldwide [3]. 

### 3.1. Data Exposure Risks

Data sharing and data security can raise different types of risks, and the main cause of these risks is when data is disclosed or exposed. One of the major consequences is that it has a negative economic effect (i.e., economic losses). Public exposure of some type of data (i.e., data indicative of welfare issues) could result in negative press for a farm and even for the industry, potential legal action within existing abuse or neglect legislation, and could lead to commercial consequences, such as the loss of company driven incentives or reduction in consumer base. Likewise, public exposure of production or economic data may affect the qualification for financial tools and incentives (e.g., loans). Exposure to other entities (e.g., business) could cause disruption in the market prices—more specifically, land and rent prices. An example of this latter exposure was a strategic data leak that benefited a company that rented out farmland. This company was leaking production data (without the consent of the farmers), which led to an increase in the rent prices [3]. These scenarios raise questions about the unethical and illegal practices (i.e., selling user data without consent), as mentioned by the Data Fairness [12]. Hence, the risk is a reasonable concern, and it is even more so when we talk about competition among countries with theoretically different standards. 

Exposure of data to other farmers may also cause some competitive risk; however, this risk is comparatively low and would result in lesser and more indirect consequences. If the data of a successful farm is sold or leaked to the competitors, this could reduce the competitive advantage of the farm relative to others: production rises in farms accessing the data, milk supply rises relative to demand, and suddenly, this has a negative impact on the market (e.g., milk price starts dropping).

These risks also raise some questions, such as how is the farmer protected? What is the legal risk of sharing the data? What could go wrong if the farmers’ data is not appropriately protected? Some type of confidentiality (i.e., anonymization or of data) of the farm-level data would be useful in providing producers a level of protection if the data is made publicly accessible or is aggregated. Data anonymization is part of all the four data codes of conduct. However, it has been suggested that data anonymization alone may provide inadequate protection given recent advancements in reidentification techniques [24]. Nevertheless, some types of data can be difficult to anonymize; for example, data for genetic evaluations, where it is necessary to know which animal is which, and the relationships amongst all animals thereby removes any anonymization. Therefore, the process of data anonymization must be done according to the type of data or the final usage of the data.

### 3.2. Transparency and Terms and Conditions

One of the most important aspects in discussing data sharing and data security is the transparency of the terms and conditions of the contract with the new technology or product. However, terms and conditions are often such lengthy and complex documents that most users do not take the time to read the terms of service and privacy policies. This explains why the phrase “I agree/read the terms and conditions” has been described as one of the most prevalent lies on the internet [25,26]. It has been reported that 97% of the people agreed to the terms and conditions without reading them [26,27], which is in line with the findings of the Dairy Brain Survey, where 92% of the respondents rarely read the terms and conditions; among these, 24% never read them (unpublished data). In addition, people that said they had read the terms and conditions reported reading times considerably below (73 s) the time needed to completely read them (29–32 min) [26]. This demonstrates the logistical burden of simply reading terms and conditions that are extremely long. For example, if we assume an average of 240 words per minute as the reading speed of an adult, reading the terms and conditions of some of the apps we normally use or know are as follows: (1) Netflix: 11:00 min, total word count: 2628; (2) Amazon: 14:12 min, total word count: 3416; and (3) Zoom: 30:12 min, total word count: 7246 [27]. It has been estimated that the average American will need 10.42 days or 250 h to read thoroughly the online terms and conditions of the apps they use [25,27]. Farmers are not the exception; are they reading the full agreements before signing them? If they do read them, do they understand all the implications? We can agree that most of the responses to this question are going to be no. Terms and conditions are frequently written in small captions, making them difficult to read, in addition to being hard to understand. One simple suggestion by the Australian Farm Data Code is that they should be written in plain language [10] and with a clear explanation of the obligations, data sharing purposes, and any benefits [7].

### 3.3. Privacy Protection

To guarantee the adequate privacy and security of the farm data, it is important to homogenize the standards of data and technology or anything that is potentially accessible to the outside world. Who should be responsible for data security and privacy once the data has left the farm? Are there scenarios in which the potential for security laps falls on the farmer, the tech company, the internet provider, or multiple separate stakeholder entities? What would be the enforcement mechanism? Should there be a fine, other legal sanction, or some other consequence if the data gets leaked? How can we provide incentives (positive or negative) in the requirement to keep the farmer’s data private? Current regulations that cover farm data do not provide consistent standards of security, meaning that the level of risk to which the end user (farmer) is exposed is at the sole discretion of independent companies, who convey that risk level through lengthy jargon-filled documents. A federal regulation would provide a level of national standardization and could provide a critical safety level for producers. 

Large datasets created by the aggregation of data from multiple sources represent a potentially valuable resource for improving farm efficiency and economy on a large scale, but some producers are often reluctant to allow their data to be incorporated in this way. To incentivize the farmer to be part of the development of these large datasets, is it enough if we agree that the farmer owns the data? That the farmer is aware of potential data security risks and can opt in or out of a sharing program? In principle, we recognize that the development of these larger datasets is very helpful to the dairy and farming industry altogether. However, we need to solve the dilemma for the individual farmers to incentivize greater participation. Are farmers entitled to some form of economic compensation for their data, such as discounts or monetary payments? In such a situation, it would not be a mutually exclusive setup where only one party benefits but more of a symbiotic, win–win situation. 

It is known that the utilization of commercial software is beneficial for farm management and that this benefit is a primary driver of technology implementation at the farm level. However, do these commercial softwares give a choice to the farmer? Are the agreements for the use of these products clear and understandable to the farmer and clearly indicate what the company’s rights over the data might be? What are the standards about data sharing with the company? Are multiple choices presented to the farmer before buying the product? The need for transparency is key, so farmers can know what they are buying and what the expectations are for handling the data. As CEMA (Comité Européen des groupements de constructeurs du machinisme agricole-European Agricultural Machinery Industry Association) [11] acknowledges, farmers and the industry are eager to share data and build a strong relationship; however, this will only be possible if the benefits and risks are well-understood and if the relationship is built with trust [7].

## 4. Existing Approaches and Possible Solutions

Fortunately, there is no need to reinvent the wheel. Precedence exists in other sectors, and existing laws could be helpful to adapt and implement those concepts into the dairy sector. Examples of successful applications and standards include the anonymization and legal protection of personal health information as described by the Health Insurance Portability and Accountability Act of 1996 (HIPAA) [28], which is a US federal law that created national standards to protect sensitive patient information from being disclosed without the patient’s consent. Adapting a similar law that clearly establishes the standards for anonymizing data, as well as the consequences of failure to do so, might be a starting point to deal with the legal risk or, at least, to provide legal recourse if the data is stored or accessed inappropriately. Another example in the US is the Federal Information Security Management Act of 2002 (FISMA) [29], which acknowledges the importance of cybersecurity practices and sets standards to ensure the integrity, confidentiality, and availability of system-related information. Any person or entity planning to work with the Department of Defense, for example, must certify that the electronic systems to be used in this work meet the FISMA standards of security. 

### 4.1. Addressing Data Governance: Farmers Bill of Rights 

All existing agricultural codes of practice are voluntary. In order to establish trust and promote participation in a data-driven economy, there is a need for a governmental effort that can help regulate, shape, and standardize data governance and data stewardship. To that end, we propose the development of a Farmers Bill of Rights that establishes and guarantees the balance of the relationship between the farmers and the industry. Some key principles of the Farmers Bill of Rights could be that the farmer owns any data obtained directly from their farm. As such, data consumers have an obligation to clearly communicate how that data is being used. Additionally, farmers have the right to exclude their data from any derivative products. Moreover, the Bill would mandate that any use or redistribution of the data explicitly state, whether these actions are performed in a properly aggregated and anonymized form or if it is identifiable to some level (farm, county, state, etc.). 

The European Union’s data privacy law (General Data Protection Regulation, GDPR) [30] and the California Consumers Privacy Act (CCPA) [31], by definition, applies to personal identifiable information about human beings and is not applicable to farm data. Nonetheless, some interesting features of the GDPR and the CCPA could be used as part of the Farmers Bill of Rights. One of the features, for example, is the right to be forgotten, so someone can literally demand that their data be removed. Similarly, the Bill could include revocable data use permissions for different data streams generated from and controlled by the farmer. Additionally, the farmer would have access to know what information is being collected by different entities and how that data is being used and shared. 

Another important aspect regarding data privacy and protection is the concept of a right or requirement to report wrongdoing. In addition to the anonymization, in the HIPPA [28], medical practitioners are required to report issues above a certain level for a patient welfare event if their parents and other controllers have access to their data and do not want it reported. It is worth considering whether there needs to be a similar standard incorporated into the Famers Bill of Rights, through which data that indicates wrongdoing gets automatically reported by companies. Conversely, significant thought should be given to the potential consequences of including such strong governmental oversight on product commercialization, innovation, and costs to the industry.

What is the fate of datasets being generated by different technological products and different companies? Are there possible restrictions to aggregate or combine different data sources? Are these databases public? Or is it fine for them to create proprietary databases? The farmer contributes data, gets the product, and buys the license, but data storage has a cost associated with it, as well as potentially added value of the data once it is processed. Addressing these questions could potentially be another aspect of the Farmers Bill of Rights for agricultural technology. Aggregation of the data generated by individual precision systems represents a potential source of highly beneficial insights, but this data often lacks the capacity to be combined across systems. Encouraging the participation of the farmer and various companies, as well as cooperation between the groups to contribute to data integration, is a key hurdle in the evolution of the dairy industry. The voluntary aggregation of data presents a business risk; producers and companies likely see the creation of compatible data streams as risks to an economically competitive future. As such, there must exist a business model that allows companies to operate. The integrative capacity of data should be encouraged, but this should be balanced with some limitations on access to the data sources and models derived from farm sources. The extent of allowable access by companies and third parties that would achieve both goals is not yet clear. It has been suggested that an open collaborative approach as the business model will be the best approach [20]. However, one example of avoiding this dilemma altogether is Article 4 of the CEMA regulation, which prohibits localized data storage, as it can constrain innovation and impact worldwide data economies [11].

Incentivizing farmers to share their data when implementing a new technology is increasingly important, as producers develop more knowledge and awareness of the issues surrounding these topics. One possible way to implement this incentivization could be that the default is to opt out, and farmers need to specifically opt in to allow their data to be incorporated into data products and gain some added benefits in the future, such as access to more information, models, or discounts, among others. This would increase the pressure on the manufacturer to clearly explain what is happening with the data and make it worth the farmers’ while. A balance between the companies and the farmers needs to be found, a hybrid model; a win–win situation for both parties is required. Part of this mutually beneficial equation lies in reducing the information imbalance described above, and producers will likely be more motivated to participate if the data sharing relationship is transparent and easily understood. This critical factor is recognized by the Australian National Farmers Federation in their recommendations [13]: “[to] encourage the fair and equitable collection, use and sharing of farm data in a way that benefits farmers and Australian agriculture; [to] build trust and confidence... in the way farm data is collected, used and shared so that, where appropriate, farm data can be utilized in ways that bring benefits to Australian agriculture”.

The proposed rights afforded by this Bill are likely to be technically challenging and unpopular with the technology companies, but again, given the limited amount of bargaining power available to individual farmers, such rights are not currently prioritized. Due to this cost of implementation and the relative imbalance in bargaining power, such changes to better represent the interests of the farmer with regards to their data is not likely to happen voluntarily and would require some state-sponsored legislation to occur in any significant fashion. At this point, we consider that a nondisclosure agreement might not be enough for convincing the farmers that their data are safe. Only through legislation that includes real consequences can you ensure access to the appropriate protections and security necessary for agricultural data.

We believe that this Farmers Bill of Rights could be among the possible solutions to address data governance issues from the highest level (Table 1). A similar approach—a federal regulation of the data practices of companies that collect agricultural data—has been suggested by others as well [24]. The Farmers Bill of Rights is a description of the legislative solution in the same way that HIPAA might be seen and a patient’s Bill of Rights or GDPR might be seen as the data subject Bill of Rights. However, there will need to be leadership and buy-ins from all stakeholders if it is to succeed. If successful, this approach will help to ensure an equilibrium of power for both parties (i.e., farmers and industry). 

### 4.2. Other Possible Solutions for More Specific Challenges

To help increase the transparency and understanding around terms and conditions, one solution is to have a summarized version of the contract available to the end user. This summarization could potentially even be automated with the use of machine learning algorithms [25]. Machine learning techniques can be used to summarize documents, detect vague language, and identify choices provided (i.e., output choices), among others. There are some tools under development that demonstrate the potential use of machine learning techniques to facilitate the understanding of the terms and condition and possibly create a short version with a key concept from the contract, avoiding bias (see Usable Privacy (https://usableprivacy.org, accessed on 14 July 2021) and Polisis (https://pribot.org/polisis, accessed on 14 July 2021)). This technique could potentially be useful to have specified standardized agreements as part of the Farmers Bill of rights by establishing term and condition deviations and summarize in plain language the terms and conditions. 

A blockchain, which is a type of database, is another tool that could help address more specific challenges, such as data privacy, data security, and data localization, as the use of a blockchain means that data is not stored on any centralized location, and access to the data can be closely monitored. However, there is no perfect tool. The inability to change, modify, or delete data once it is stored on the blockchain could be an advantage and disadvantage at the same time. It could be an advantage, as nobody can alter or change the data; however, with some of the possible opt-out options, it will be impossible to delete the data that was collected [32]. From the environmental perspective, the concern is due to the large amount of energy needed that is associated with carbon emissions [33]. The Cambridge Centre for Alternative Finance (CCAF) [34] estimates that the current annual estimate of energy consumption of Bitcoin, which uses a blockchain, is 50 terawatt/hour (TWh), which could satisfy the energy need of the University of Cambridge for 365 years, is around 0.55% of the global electric production, and is equivalent to the annual energy consumption of countries as Sweden or Malaysia. Currently, the dairy industry blockchain is used in food supply chain management systems. Another possible complementary solution to deal with data privacy and data security is federated learning [5,35,36,37]. Federated learning was introduced by Google AI in 2017 [35] and gives a simple description on how it works with respect to mobile phones: “It works like this: your device downloads the current model, improves it by learning from data on your phone, and then summarizes the changes as a small, focused update...all the training data remains on your device, and no individual updates are stored in the cloud.” Therefore, it is an approach to train machine learning algorithms in multiple local devices (i.e., decentralized devices) without exchanging data among them, trying to preserve the data privacy. However, there are still concerns about data privacy, based on the provenance of the data (i.e., sensitivity of the data) and on the granularity of what is defined as data privacy [38].

## 5. Conclusions

There are multiple factors that make the data governability in dairy farming a complex issue. The goal of this commentary paper is to spark a discussion of some of the challenges and their possible solutions. We are aware that this paper is not fully inclusive or comprehensive and that there are additional issues not addressed here. However, our motivation is to engender a thoughtful discussion of the problems and possible solutions. We propose as a first great step working towards developing and implementing a Farmers Bill of Rights rooted in some key points: (1) producers own the data generated on their own farm, (2) data security and transparency are fundamental; however, they only matter if there are different choices (e.g., an opt-out option) in the contract, and (3) a method of incentivizing farmers is required that encourages them to be comfortable sharing their data in order to better enable a data economy within the dairy industry. In addition, the concept idea could be used or followed by other farm industries and could be a good guideline for other countries around the world. 

## Figures and Tables

**Table 1:** Data governance issues and their possible solutions addressed by the proposed Farmers Bill of Rights.

Current Data Governance Problem	Possible Solution Addressed by the Farmer Bill of Rights
Data risks: disclosure or exposure of data	Data anonymization according to the type of data or the final usage of the data
People do not read terms and conditions, because they are too long and difficult to understand	Have specific and standardized agreements by establishing terms and conditions deviations, which should be summarized in plain language
Farm data privacy and security	A federal regulation would provide national standards for cybersecurity practices to ensure the integrity, confidentiality, and availability of system-related information
Data sharing	Promote a data sharing relationship based on transparency and easy understanding Access to know which data is being collected and how the data is being used and shared Give farmers options to opt in or out, to revoke data use permissions, right to be forgotten, etc.

## USDA Co-Author Disclaimers

The views and opinions expressed in this review do not necessarily reflect the official policy or position of the authors’ respective employer or government. The mention of any trade name, proprietary product, or specific equipment does not constitute a guarantee or warranty by USDA-ARS and does not imply its approval to the exclusion of other products that may also be suitable. The USDA-ARS is an equal opportunity and affirmative action employer, and all agency services are available without discrimination.
